# Fairness-Oriented Semichaotic Genetic Algorithm-Based Channel Assignment Technique for Node Starvation Problem in Wireless Mesh Networks

**DOI:** 10.1155/2021/2977954

**Published:** 2021-08-09

**Authors:** Fuad A. Ghaleb, Bander Ali Saleh Al-Rimy, Wadii Boulila, Faisal Saeed, Maznah Kamat, Mohd. Foad Rohani, Shukor Abd Razak

**Affiliations:** ^1^School of Computing, Faculty of Engineering, Universiti Teknologi Malaysia, Johor Bahru 81310, Malaysia; ^2^Department of Computer and Electronic Engineering, Sana'a Community College, Sana'a 5695, Yemen; ^3^RIADI Laboratory, National School of Computer Sciences, University of Manouba, Manouba 2010, Tunisia; ^4^College of Computer Science and Engineering, Taibah University, Medina 42353, Saudi Arabia

## Abstract

Wireless mesh networks (WMNs) have emerged as a scalable, reliable, and agile wireless network that supports many types of innovative technologies such as the Internet of Things (IoT), Wireless Sensor Networks (WSN), and Internet of Vehicles (IoV). Due to the limited number of orthogonal channels, interference between channels adversely affects the fair distribution of bandwidth among mesh clients, causing node starvation in terms of insufficient bandwidth distribution, which impedes the adoption of WMN as an efficient access technology. Therefore, a fair channel assignment is crucial for the mesh clients to utilize the available resources. However, the node starvation problem due to unfair channel distribution has been vastly overlooked during channel assignment by the extant research. Instead, existing channel assignment algorithms equally distribute the interference reduction on the links to achieve fairness which neither guarantees a fair distribution of the network bandwidth nor eliminates node starvation. In addition, the metaheuristic-based solutions such as genetic algorithm, which is commonly used for WMN, use randomness in creating initial population and selecting the new generation usually leading the search to local minima. To this end, this study proposes a Fairness-Oriented Semichaotic Genetic Algorithm-Based Channel Assignment Technique (FA-SCGA-CAA) to solve node starvation problem in wireless mesh networks. FA-SCGA-CAA maximizes link fairness while minimizing link interference using a genetic algorithm (GA) with a novel nonlinear fairness-oriented fitness function. The primary chromosome with powerful genes is created based on multicriterion links ranking channel assignment algorithm. Such a chromosome was used with a proposed semichaotic technique to create a strong population that directs the search towards the global minima effectively and efficiently. The proposed semichaotic technique was also used during the mutation and parent selection of the new genes. Extensive experiments were conducted to evaluate the proposed algorithm. A comparison with related work shows that the proposed FA-SCGA-CAA reduced the potential node starvation by 22% and improved network capacity utilization by 23%. It can be concluded that the proposed FA-SCGA-CAA is reliable to maintain high node-level fairness while maximizing the utilization of the network resources, which is the ultimate goal of many wireless networks.

## 1. Introduction

Wireless mesh networks (WMNs) enable flexible, robust connectivity and are a means to various applications, such as healthcare, smart grids, Internet of Things (IoT), Internet of Vehicles (IoV), and intelligent transportation systems [1, 2]. The ability to use different radio technologies, including IEEE 802.11 (a/b/g/n) and 802.16, makes WMN flexible enough to support many manufacturing standards for wireless networks [3–6]. Client meshing is one of the important characteristics that distinguish WMNs from conventional wireless ad hoc networks [7–12]. WMNs improve the development of many fields such as routing protocols [8, 13–16], media access control [17, 18], and energy conception [19], among many others [3, 7, 20]. WMN nodes are characterized as dual-functioning, such that they play both client and router roles by automatically establishing and maintaining connectivity among themselves [4]. As opposed to Point-to-Point (PTP) communication that is used by traditional ad-hoc networks, WMNs use multipoint to multipoint (MTM) communication to increase network scalability, reliability, and capacity by enabling a mesh node to communicate with more than one other mesh node simultaneously [21]. Such property contributes to achieving reliable, low-maintenance, low-cost, and robust mesh networks. A WMN consists of three main components, namely mesh routers, gateways, and clients [4]. Mesh routers work as a backbone that connects mesh clients and gateways. Gateways are mesh nodes that interconnect the WMN with other networks and services such as the Internet, data centers, and servers. Mesh clients are nodes that end-users use to connect to the WMN. These nodes may be laptops, mobiles, vehicles, health care appliances, and any other IoT devices [22–27]. A mesh client reaches the resources or services by connecting to WMN via mesh routers, which, in turn, redirects the traffic from/to the gateway. Mesh routers utilize multiple radio interfaces with multiple channels by using multiple radios to decrease the interference between colocated communication links and improve the throughput, connectivity, and capacity of the network. To decrease such interference, the colocated links need to use nonoverlapping (orthogonal) channels. However, the limited number of orthogonal channels allocated in the wireless standards makes the interference between adjacent links inevitable. Therefore, effective channel assignment is key to ensure high network throughput, connectivity, and capacity [3, 7, 28].

Channel assignment algorithms play an important role in improving the connectivity, throughput, and capacity of WMNs. These algorithms aim at finding an optimal distribution of the channels among the colocated links to maximize the utilization of network resources and reduce interference, that is, a channel assignment algorithm that tries to improve the bandwidth utilization by reducing the interference and efficiently utilizing the frequency spectrum [3, 4]. The problem of channel assignment is usually formulated as a graph coloring problem, which is naturally an NP-hard (a nondeterministic polynomial-time) problem whose optimal solution might not exist [29]. This is because, in most practical situations, there may be insufficient orthogonal channels to ensure interference-free channel assignments. A large number of mesh devices may share a single common channel to reduce network interference [30, 31]. However, increasing the number of adjacent devices that use the same channel increases the collisions and adversely affects the network performance. A large collision wireless domain leads to collisions that affect the connectivity, bandwidth, and capacity of WMNs. In addition, unfair channel distribution among mesh links causes node starvation problems.

**Figure 1:**
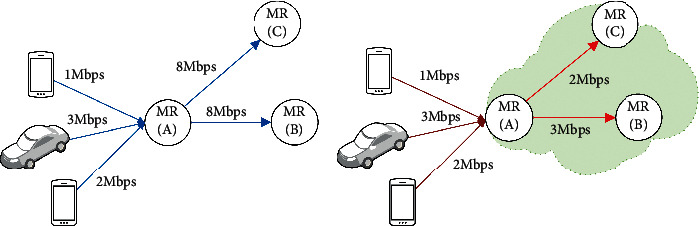
Node starvation problem. (a) Nominated capacity (ideal scenario). (b) Capacity after channel assignment (node starvation problem).

The node starvation problem occurs when the surrounding links are unable to support the required bandwidth of the adjacent clients due to link interference.  Figure 1 illustrates an example where the node starvation problem occurs. As shown in Figure 1(a), the capacity in terms of the total supported bandwidth of mesh router A is 16 Mbps while the required bandwidth is at 6 Mbps. In the ideal situation, the available bandwidth can satisfy the requirements of mesh clients. However, Figure 1(b) shows that mesh router A is unable to support more than 3 Mbps bandwidth after channel assignment due to interference among adjacent links. This results in the node starvation problem. Thus, a fair channel assignment algorithm should guarantee the equitable distribution of the bandwidth among the links such that all clients are served fairly. Therefore, a fair channel assignment should aim at ensuring that all links in the network can achieve a data rate that is suitable for all relevant nodes. Hence, the data rate of each link after the channel assignment should be consistent with the designated data rate of the link.

Over the last decade, many solutions have been suggested to address various channel assignment problems [3, 13, 21, 32–40]. However, there is no feasible deterministic solution to find the optimal solutions in a finite amount of time due to the NP-Hard nature of the channel assignment problem. Existing solutions used heuristics algorithms to approach the channel assignment problem. Unfortunately, heuristic algorithms may lead to inefficiencies, whereby the appropriate solutions may not be found within a reasonable period due to being trapped in local minima causing the unfair distribution of the channels in the network. Metaheuristic algorithms such as genetic algorithms and Tabu search address this shortcoming by approaching the global minima using the concept of natural selection and also the evolutionary theory [3, 37, 39–42]. That is, a new solution is resulting from combining two good solutions. However, the existing genetic algorithm- (GA-) based channel assignment algorithms [37, 39–42] have overlooked the fairness issue. Most of those algorithms try to minimize the total sum of the link interferences, which, unfortunately, does not guarantee fair channel distribution and cannot prevent node starvation problems. Although fair channel assignment was the subject of several studies in recent years [2, 32, 33, 43–46], most of those solutions use heuristic approaches. In addition, many of those approaches focus on flow fairness, whereby the channel assignment algorithm distributes the available nonoverlapping channels in such a way that flows (paths) in the networks have equal data rates. On the other hand, node fairness, which can significantly improve fairness, has received low research attention [1, 3]. Node fairness can be achieved by equitable distribution of bandwidth over the nodes. Consequently, equitable distribution of the channels should make each node end up with the desired bandwidth. In Figure 1(b), for example, node A requires a minimum bandwidth of 6 Mbps, but it is given only a total of 3 Mbps. Ignoring node fairness leads to node starvation, which adversely affects the capacity and throughput of WMNs.

To this end, this paper proposes a Fairness-Oriented Semichaotic Genetic Algorithm-Based Channel Assignment Technique (FA-SCGA-CAA) that addresses the issue of node starvation through fair (equitable) channel distribution among mesh nodes in the WMN. The channel assignment problem has been formulated as an optimization problem with two objectives, minimizing the interference and maximizing node fairness. Because interference and fairness are not linearly correlated, this study introduces a new nonlinear fitness function that aims at minimizing the interference while maximizing the bandwidth utilization to ensure fair distribution of the nonoverlapping channels and guarantee the required bandwidth for each mesh client. FA-SCGA-CAA optimizes fairness based on multiple criterion using a modified version of the genetic algorithm (GA). The modification includes proposing a semichaotic technique for creating the primary chromosome with powerful genes. Such a chromosome was used to create a strong population that directs the search towards the global minima effectively and efficiently. The outcome is a nonlinear fairness-oriented fitness function that aims at maximizing the link fairness while minimizing the link interference. The contribution of this paper is four-fold:

1. The Fairness-Oriented Semichaotic Genetic Algorithm-Based Channel Assignment Algorithm (FA-SCGA-CAA) is proposed to maximize link fairness while minimizing link interference.
2. A semichaotic genetic-based technique is proposed to create a diverse population with informative features that converge at the best solution and avoid being trapped in the local minima. The semichaotic technique is proposed to address two main issues of genetic algorithms, which are to speed up the convergence process of the algorithm and to increase the diversity of the searched solutions to find the best feasible solution.
3. The problem of fair channel assignment is formulated as an optimization problem that entails a fitness function that combines several factors representing the network topology, link capacity, and required bandwidth/throughput to minimize link interference while maximizing link fairness.
4. A new nonlinear fitness function is proposed to integrate both interference and fairness in one fitness function for minimizing link interference while maximizing link fairness that is directly reflected in improving client fairness.

## 2. Related Work

The channel assignment problem in a multiradio wireless mesh network has been the subject of many recent studies [3, 13,21, 33–38, 47]. Many methods were used in those solutions such as graph-based [48, 49], optimization-based [37, 45, 50], and artificial intelligence-based [37, 39, 40, 51] techniques. A detailed review of those methods can be found in the following surveys [3, 7, 52]. Most of those studies aimed at minimizing global network interference. The link interferences were estimated using either protocol [21, 34] or physical interference [53] models. The hypothesis behind those solutions was to reduce global network interference leading to improving the utilization of network capacity, throughput, goodput, and delay, among other desired characteristics [3, 7, 52]. Although many of these solutions minimized global interference and accordingly improved the performance of the network, such solutions suffer from both links and node starvation problems due to the unfair distribution of the channels among the links. Nodes' starvation problem occurs when a node tries to use a link with high interference. To solve this issue, a fair distribution of the nonoverlapped channels is required. Fair channel distribution has been the focus of several recent studies [2, 32, 33, 36, 43–46, 54–57].

Fairness is defined by many researchers as the equal distribution of the resources among equal nodes [2, 43]. This broad definition of fairness has led to many open challenges in channel assignment algorithms that need to be addressed such as fair bandwidth [56], fair throughput [58], fair access to the channel [56, 59, 60], load balancing, and energy balancing [61]. However, the interference among communication channels is a major challenge that impedes the fair utilization of WMN resources and causes node starvation problems. Fairness in wireless networks can be categorized based on several criteria such as granularity, time, resource type, and access mode. From the granularity perspective, fairness can be categorized into system-wide fairness [36, 62], per-flow fairness [43, 55], per-link-fairness [33, 46], and per-node-fairness [36, 43]. Such categorization determines the level at which fairness is achieved. System fairness is viewed from the perspective of the whole system, which is achieved when all mesh nodes attain individual fairness [36]. Per-flow fairness refers to equal bandwidth for all traffic flows arriving at the gateway [55], which is achieved by assigning nonoverlapping channels to the links before allocating those links to flows based on the interference model [43]. Per-link fairness-based solutions aim at ensuring equal distribution of the bandwidth among links. The per-link fair channel allocation tries to prevent node starvation phenomena that could happen due to the interference. Therefore, the solutions that try to achieve per-link fairness aimed at minimizing the interference among links. Per-node fairness-based solutions aim at ensuring that all nodes in the network have obtained equal access opportunity to the network. This can be achieved if the channel assignment algorithm takes the traffic demands of each individual link as a requirement during channel assignment to achieve fairness and prevent node starvation problems. This paper focuses on per-link fairness in order to achieve the per-node fairness. To the best of our knowledge, such granularity of fairness has not received enough investigation yet.

In their study, Qu et al. [36] proposed a channel assignment algorithm to prevent the flow starvation problem. An interference model was embedded into the channel assignment algorithm to better estimate interference on the links to eliminate border effect and flow starvation. However, the algorithm was designed for single radio mesh networks. Moreover, such a solution does not consider node starvation as it aims only to achieve per-flow fairness.

Bakhshi and Khorsandi [46] used integer linear programming (ILP) to develop a dynamic channel assignment algorithm to achieve flow fairness. However, the solution defines fairness as a function of the number of accepted demands with source-destination pairs over the specific threshold. Hence, fairness is achieved only when the number of transmission demands approaches a particular number. This approach can improve network capacity if the number of transmission demands is known before channel assignment. However, this approach is scenario-specific and dynamic, and it cannot be defined in advance. Beheshtifard and Meybodi [34] devised an adaptive scheme based on learning automata to maintain channel assignment when network traffic demands dynamically change. However, the scheme lacks a mechanism that ensures fair allocation of the channels among links.

Ghaleb et al. [33] proposed a channel assignment algorithm based on weighted link ranking to achieve an equitable distribution of the orthogonal channels. The equitability (fairness) was achieved by employing multiple criteria like proximity from the gateway, expected traffic, and link capacity, to rank the mesh links. Then, the nonoverlapping channels were assigned accordingly. However, such an approach lacks proper fairness measures that can effectively evaluate the node fairness achieved. Al-Rimy et al. [32] proposed a channel assignment scheme that considers user mobility and fairness. The scheme adaptively adjusts to channels assignments according to the dynamic change in the network due to user mobility. The scheme uses the multirotational channel assignment algorithm that was proposed by Ghaleb et al. [33] to achieve fairness which lacks proper fairness measures.

In their study, Liu et al. [44] proposed a genetic-algorithm-based routing algorithm combined with a channel assignment technique that aims at maximizing the minimum flow rate to improve flow fairness. However, maximizing the number of flows does not guarantee that node starvation will be prevented due to the inconsideration of per-link interference during channel assignment. The genetic algorithm was used to search for the chromosome with the highest number of flows. In their solutions, the chromosomes were represented and evaluated based on the power level of the channels. However, power level has spatiotemporal characteristics that lead to unstable and short-term channel assignment and, thus, such solution neither prevents link starvation nor flow starvation.

To sum up, many recent studies investigated fair channel assignment solutions. However, there are two main drawbacks of the extant research, which can be described as follows. First, most of those studies simply minimize the total interference in the network to achieve effective utilization of network resources. Such solutions lead to fair starvation of nodes and links which is not the goal of fairness in channel assignment. The common hypothesis among these solutions states is that reducing the total interference can improve network performance, which directly achieves fairness among mesh nodes. This hypothesis is inaccurate because reducing the total interference does not necessarily ensure equal distribution of channels in the network. In addition, fairness was represented in terms of total power level, total interference, or total throughput of the network. Moreover, the extant studies aimed at equal distribution of network resources (such as the bandwidth) among nodes, which does not necessarily lead to fairness and thus does not prevent node starvation problem. The equitable distribution of the resources among all nodes is the ultimate goal of a fair channel assignment algorithm to resolve the node starvation problem. That is, equal nodes only should receive equal resources. The resources should be distributed based on the requirements, which are not necessarily equal. The second drawback of the existing solutions is the use of heuristic methods to assign the channels. Unfortunately, heuristic techniques are not appropriate solutions as they are scenario-dependent and thus cannot be generalized. Metaheuristics techniques on the other hand such as genetic algorithms seem to be promising to approach channel assignment algorithm in WMN due to the smaller search scope as it searches in a controlled population sample. However, randomly generating the population samples may lead to a premature solution since the search can be directed to local minima. This study addresses this issue by proposing a fair channel assignment algorithm with more granularity. Links and nodes fairness have been represented based on the impact of interference on their expected bandwidth. Thus, a fairness-oriented fitness function was proposed and integrated into the genetic algorithm-based channel assignment, which aims at maximizing node and link fairness, while minimizing interference.

## 3. Materials and Methods

**Figure 2:**
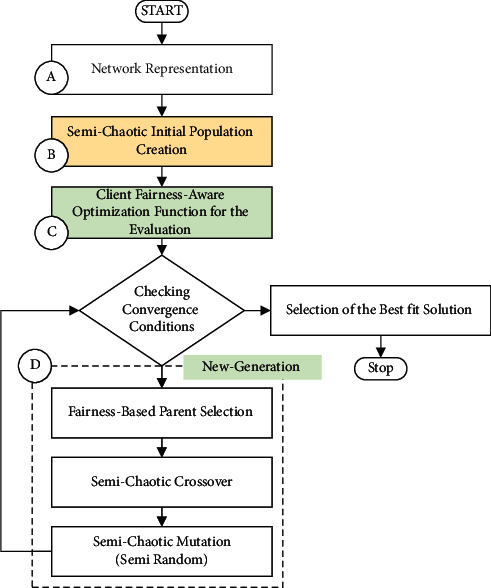
Flow chart of the suggested approach.

In this study, the problem of channel assignment is formulated as an optimization task that maximizes link fairness to avoid node starvation problems. Thus, we have proposed a semichaotic genetic algorithm to solve such a problem. The proposed algorithm aims at finding the most effective solution that achieves node fairness and addresses the link starvation problem. The genetic algorithm (GA) is a metaheuristic algorithm inspired by the evolution theory and natural selection. GAs are adaptive search techniques that can find the optimal global solution by manipulating and recursively generating a new population of solutions from an initial population space [42]. GA is used for combinatorial optimization problems, namely the NP-optimization problems (NPO) since they search from one population of points in search space to another and tend to focus increasingly on areas with deeper minima [31, 32, 42, 63]. The proposed algorithm comprises five steps as follows: network representation, semichaotic-based initial population creation, fairness-aware individual evaluation, parent selection, and children generation or offspring. The channels are represented by genes. Hence, the number of genes is equal to the number of nonoverlapping channels. Therefore, the chromosome represents a solution, which is a series of channels (genes) assigned to the radios in the network.  Figure 2 shows the flowchart of the proposed algorithm.

As shown in Figure 2, there are four main stages in the algorithm. The first stage is network representation. The WMN is represented as a graph to represent the interference between the links as elaborated in Section 3.1. Then, the initial population has been created by developing a new semichaotic method, in which the genes of the primary chromosome are created using a multicriterion link ranking-based channel assignment algorithm [11]. In doing so, the initial population inherits powerful features from the father (the primary chromosome). The multicriterion links ranking-based channel assignment algorithm is described in Section 3.2. To create diversity in the population and prevent the convergence to a local minimum, a chaotic-based algorithm is used to create the initial population. Next, the individuals in the population are evaluated using a novel nonlinear fitness function. The optimization objectives are fuzzed into one representative nonlinear function and the so-called fairness-aware interference minimization function. This function correlates the interference to fairness by formulating the link fairness as a function of interference and the data rate. Thus, individuals or chromosomes are evaluated according to fairness conditions. That is, if an individual is found to have an acceptable fairness index, the objective is achieved, and the algorithm is stopped. Otherwise, the algorithm continues to the next step. A detailed description of this function is found in Section 3.3. The next stage is the selection of the parents for the new generation. The best individuals with high values of fairness are selected for the offspring. Then, the semichaotic process is followed to create a new generation from the selected parents' chromosomes. For creating the children of the new generation, a semichaotic mutation approach is developed in which the genes (channels) with high fairness values are chosen for crossover, while the genes with low fairness are replaced using the semichaotic process. Finally, to maintain the diversity among the new generation, the genes with high fairness values are fixed while the genes with low fairness values are mutated. This stage is iterated until the convergence in the global minima is obtained, or the maximum number of iterations is reached.  Section 3.4 elaborates this stage in more detail.

### 3.1. Network Representation

**Figure 3:**
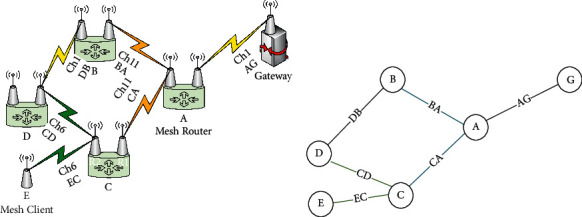
Network diagram represented as a graph. (a) Mesh network. (b) Graph representation.

The wireless mesh network (WMN) is represented by a graph that can represent the interconnection of all nodes connected in the network (see Figure 3). The graph consists of two main components, namely vertices and edges (Figure 3(b)). A vertex can be a mesh router, mesh client, or mesh gateway. Meanwhile, the edges represent the links between the vertices (e.g., the link AG in Figure 3(b)). A wireless link can be defined as a dedicated connection between two radio interfaces on two different nodes. The radio interfaces that form the links share the same radio characteristics, such as channel frequency, bandwidth, speed, and encoding. A link length is the Euclidian distance between the positions of the radio interfaces that form that link. Two links are considered conflicted (and can also be partially overlapped) if they use the same channel and the distance between them is less than the channel interference range (see Link BA and CA Figure 3(b)). The data rate of the links is affected by two factors: the amount of interference and the link length. A good channel assignment algorithm can result in low interference (internal interference) and high fairness. That is, the interference should be reduced and distributed fairly on the network so that nodes do not starve to obtain equitable bandwidth.

### 3.2. Semichaotic Initial Population Formation

**Figure 4:**
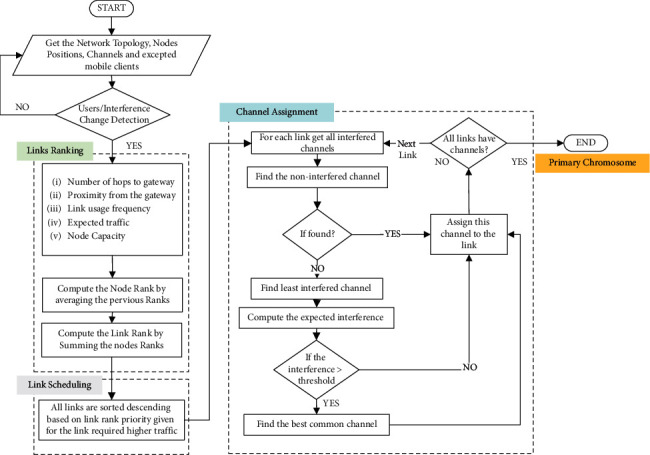
Flow chart for the generation of the primary chromosome.

Forming the initial population is an important step in genetic algorithms directly affecting the quality of the results. There are two approaches for the initialization, namely heuristic and random. The heuristic approach may direct the genetic algorithm to fast convergence to a local optimum, while the random-based approach may slow down the convergence. Therefore, in this study, a semichaotic-based population formation technique is proposed. In this technique, first, the primary chromosome which constructs the entire population has been created using a multicriterion channel assignment algorithm. The primary chromosome was created using strong genes so that the entire population inherits those powerful genes.  Figure 4 shows the flow chart of the designed primary chromosome generation technique. The multicriterion channel assignment algorithm [11] was used to create the primary chromosome of the initial population.

The multicriterion channel assignment algorithm is a heuristic algorithm that uses multiple criteria derived from network topology and the expected traffic patterns used in the proposed channel assignment algorithm. As shown in Figure 4 and the pseudocode 1 in Algorithm 1, the algorithm is composed of three main steps, namely link ranking, link scheduling, and channel assignment. In the link ranking step, five criteria are used to rank the nodes as follows: the number of hops to the gateway, proximity from the gateway, usage frequency, and capacity. The node rank is normalized, and a score for each node is given. The link rank is obtained by summating the scores of the nodes that form the links. The next step is link scheduling, in which the links are sorted in descending order. Thus, the channel assignment algorithm starts distributing the nonoverlapping channels over the links that have high ranks. Finally, the channel assignment is performed to obtain the primary chromosome to create the initial population. The process of obtaining the primary chromosome is as follows (see Figure 4 and Algorithm 1). First, for each link in the graph, the list of all interfered channels is obtained. Then, the list of all nonoverlapping channels that can be used for the link is obtained. If there is any such channel, it is assigned to the link. The algorithm then moves to the next important link. If the nonoverlapping channels are not available for the current link, the least interfering channel is assigned.

The least interfering channel is calculated in every iteration of the channel assignment. To estimate the expected interference *f*_chl_, the mesh network is represented as a conflicting graph where the vertices are the links, and the edge is the shortest distance between any two nodes from adjacent links. The link graph represents the mesh network, which is used for computing the nodes and links ranking. The interference graph is used to obtain the interfered radios which are used to form the conflicting graph. The conflicting graph is used to obtain the list of the interfered links during channel assignment. It is also used to calculate the expected bandwidth that the link can support after channel assignment. Thus, the conflicting graph is used to evaluate the effectiveness of the generated solutions. It is worth noting that the channel assignment algorithm in this stage uses a threshold for the acceptable interference for assigning a channel to a link. If the resulting interference is not accepted, the algorithm assigns a common channel as there is no other option in this case. That is, the resulting bandwidth of the link after channel assignment should not be less than the bandwidth if a common channel was used. The channel assignment algorithm continues to assign the channels until all links in the network obtain appropriate channels. Upon the completion of this stage, the primary chromosome is obtained and the initial population can be created.

The semichaotic technique is used to create the initial population from the primary chromosome. The weak genes (the link after the channel is assigned) with high interferences are replaced randomly from the available channels. Meanwhile, the strong genes (the links with nonoverlapping channels) are fixed. In doing so, two main advantages are obtained improving the metaheuristic approaches, namely the genetic algorithms for approaching the NP-Hard problems: the speed of the convergence and the effective solutions that are obtained. Because the semichaotic technique randomizes a portion of gene space in the chromosome, the search scope is small, and thus fast convergence is obtained as well as the good quality of the obtained solution.

### 3.3. Fairness-Aware Fitness Function

After preparing the initial population using the semichaotic technique, the set of chromosomes in the initial population is evaluated. This required a fitness (objective) function in the genetic algorithm. The fitness function is used to evaluate the solution domain. As stated previously, the channel assignment aims to maximize the link fairness while minimizing the link interference to maintain a suitable link data rate for the mesh clients to address node starvation problems. The objective function of the optimization is written as follows:

(1) $$\begin{array}{c} \text{Maximize}\left(\text{Fairness}\_\text{index}\right), \end{array}$$

where Fairness_index can be expressed according to Jain's index as follows:

(2) $$\begin{array}{c} \text{Fairness}\_\text{index}=\frac{{\left[\sum_{i=1}^{n}lf_{i}\right]}^{2}}{n\sum_{i=1}^{n}lf_{i}^{2}}, \end{array}$$

where *lf*_*i*_ is the fairness of the link *i*. Link fairness *lf* can be defined as a function of the link data rate as follows:

(3) $$\begin{array}{c} lf=\frac{\text{Actual}\_\text{Link}\_\text{Data}\_\text{Rate}}{\text{Require Lin Data Rate }}. \end{array}$$

The data rate needs to be maximized to achieve higher fairness. The Actual_Link_Data_Rate is the expected data rate of the link after channel assignment while the Require Lin Dat Rate is the minimum accepted data rate of the link. In other words, the following condition should be satisfied:

(4) $$\begin{array}{c} \text{Actual}\_\text{Link}\_\text{Data}\_\text{Rate}\geq \text{Required}\_\text{Link}\_\text{Data}\_\text{Rate.} \end{array}$$

The Required_Link_Data_Rate can be calculated based on the sum of the required data rate of expected mesh clients or routers. The Required_Link_Data_Rate of each link in the network is assumed to be known. This is a reasonable assumption as the number of clients that will be connected to the mesh routers can be controlled according to the available resources. The data rate is indirectly correlated with the interference; i.e. if the interference is high, the data rate is low and vice versa. The actual data rate of a link can be calculated using Shannon–Hartley theorem which states that the data rate depends on the channel bandwidth and the signal-to-noise ratio, as in the following:

(5) $$\begin{array}{c} \text{Actual}\_\text{Link}\_\text{Data}\_\text{Rate}=\text{BW}*\;\log_{2}\;\left(1+\text{SNR}\right).\; \end{array}$$

Here, BW is the bandwidth of the channel, SNR is the signal-to-noise ratio, and capacity is the capacity of the channel in bits per second. To calculate the SNR, we first calculate the noise as the difference between the power of the transmitted signals and the power of the received signal.

Thus, the signal-to-noise ratio (SNR) can be calculated as follows:

(6) $$\begin{array}{c} \text{SNR}=\frac{\left(\text{power of signal}\right)}{\left(\text{power of noise}\right)}. \end{array}$$

SNR is usually expressed in decibels (dB) as follows:

(7) $$\begin{array}{c} \text{SNR}\left(\text{db}\right)=10*\;\log_{10}\left(\frac{\text{power of signal}}{\text{power of noise}}\right). \end{array}$$

As the bandwidth can be known from the channel characteristics and SNR can be calculated as in the above equation, the data rate can be calculated. Practically the strength of the received signal is measured using a hardware sensor, given that the transmission power is known; thus, the expected or the actual data rate after channel assignment can be estimated. However, for the sake of this study, the link data rate has been estimated using a formula that is derived from the Shannon-Hartley theorem and the path loss assuming free-space path loss. Let *l*_*l*_ be the link_length, and TSS is the transmission signal strength in dB *m*. The received signal strength RSSin dB *m*  can be derived as follows: RSS =TSS – 10*∗n∗*  log_10_(*ll*),

(8) $$\begin{array}{c} \text{RSS }-\text{TSS}=–\;10*n*\log_{10}\left(l_{l}\right),\\ \text{TSS }-\text{ RSS}=10*n*\log_{10}\left(l_{l}\right),\\ \text{noise}=10*n*\log_{10}\left(l_{l}\right),\\ \frac{\text{TSS}}{\text{noise}}=\frac{\text{TSS}}{10*n*\;\log_{10}\left(l_{l}\right)}\;,\text{thus},\\ \text{SNR}=\frac{\text{TSS}}{10*n*\;\log_{10}\left(l_{l}\right)}. \end{array}$$

As the interference influences the data rate, the interference on each length is the total overlapping of the link interference index. Thus, (8) can be rewritten in terms of link_interferencence (denoted by *l*_*i*_):

(9) $$\begin{array}{c} \text{SNR}=\frac{\text{TSS}}{10*n*l_{i}*\;\log_{10}\left(l_{l}\right)}. \end{array}$$

Consequently, using this formula, the data rate can be estimated numerically. By minimizing the link interference index, the SNR will be higher, which increases the data rate as well. The link interference index (*l*_*i*_) can be estimated in terms of channel overlabing ratio (denoted by *C*_ov(*l*, *m*)_) as follows:

(10) $$\begin{array}{c} l_{i}=\sum C_{\text{ov}\left(l,m\right)},\;\forall m\in l\text{ overlapped neighboring channels}. \end{array}$$

The channel overlabing ratio can be estimated based on the conflicting graph utilizing the interference matrix that was proposed in [33]. To granularly evaluate the fairness of each link in the network, the formula in (3) was used. Then, by substitution, the link_fairness(*l*_*i*_) in the Jain's index equation which was presented in (2), the following formula can be used to compute the overall fairness. Hence, the objective function can then be written as follows:

(11) $$\begin{array}{c} \text{Fairness index}=\frac{{\left[\sum_{i=1}^{n}{\left(\text{BW}*\;\log_{2}\;\left(1+\text{SNR}\right)/\text{Required}\_\text{Link}\_\text{Data}\_\text{Rate }\right)}_{i}\right]}^{2}}{n\sum_{i=1}^{n}{\left(\text{BW}*\;\log_{2}\;\left(1+\text{SNR}\right)/\text{Required}\_\text{Link}\_\text{Data}\_\text{Rate }\right)}_{i}^{2}}, \end{array}$$

where the BW channel bandwidth and SNR is the signal-to-noise ratio, which can be estimated using (8). The genetic algorithm aims to maximize the fairness index, as presented in (11).

(12) $$\begin{array}{c} \text{Max}\left(\begin{array}{c} \text{Fairness }\\ \text{index} \end{array}\right). \end{array}$$

### 3.4. Semichaotic-Based New Generation

This stage consists of three steps as follows:

(1) *Fairness-Aware Parent Selection*. Based on the fitness function presented in (11), the individuals with the best fairness index are selected for the next population. The following formula is used to select the parents:
  (13) $$\begin{array}{c} \text{Selection rule}:\text{ if}\left\{\begin{array}{cc} \text{Fairness}\_\text{index}\geq \left|\mu +\sigma \right|, & \text{selected,}\\ \text{otherwise,} & \text{not selected,} \end{array}\;\right) \end{array}$$
where *μ* is the mean of the fairness of the population and *σ* is the standard deviation of the population fairness. Although the number of selected parents is not necessarily fixed, the number of children (population size) is fixed in every generation.
(2) *Crossover*. In the crossover process, the children are created from two parental chromosomes that were randomly selected from the previous step. The genes of the new child are mixed with the genes of the parents. The crossover process tends to pull the population towards the local maxima (the highest fairness in the population). For every two pairs, the powerful genes (high fairness link) are fixed, and the weak genes (low fairness link) are removed.
(3) *Mutation*. In the mutation step, the genes with low fairness are replaced randomly while the genes with high fairness are fixed. The mutation process aims at increasing the diversity and preventing the algorithm from being trapped in local minima.

Algorithm 1 presents the pseudocode of the proposed Fairness-Oriented Semichaotic Genetic Based Channel Assignment (FA-SCGA-CAA), and Table 1 illustrates the description of the symbol. In Algorithm 1, the algorithm takes the Network Topology *G* with Nodes Positions, Available Channels ch, Fairness Tolerance Rate *τ*, and the maximum iteration max_*i* as input. Then, it returns the best solutions in the chromosome *p*_*g*(*n*)_ which contains the links and the associated channels with the highest fairness index and lowest interference channels. As discussed in Section 3.1, the mesh network is represented by a graph to facilitate the numerical analysis of the performance of the network. The primary chromosome formation (in lines 1–8 in the pseudocode presented in Algorithm 1) and the semichaotic-based initial population creation (in lines in the pseudocode lines 12–18) are elaborated in Section 3.2. The fairness evaluation phase (see lines 20–28) was conducted using a novel fitness function (see (11) and line 26 Algorithm 1) to minimize link interference while maximizing the fairness. The fairness evaluation phase is presented in detail in Section 3.3. Natural selection was used to select the parents for the next generation (see lines 30–32 in the pseudocode). That is, the parents that have high fairness were selected. Meanwhile, parents with low fairness were neglected. The creation of the new generation phase is discussed in Section 3.4. As can be observed from Algorithm 1 and the earlier discussion in this section, the original genetic algorithm has been improved in many ways. Firstly, the primary chromosome was created using strong genes so that the entire population inherits those powerful genes. The multicriterion link ranking algorithm was used to create the strong genes of the primary chromosome (see Figure 4 and explanation in Section 3.2). Then, a semichaotic technique was proposed to create the initial population. In doing so, the initial population consists of powerful genes inherited from the primary chromosome. Accordingly, the algorithm converges fast to the best solution. Then, as shown in Algorithm 1, the pseudocode, a new fitness function, has been formulated to maximize the fairness among individuals and prevent node starvation problem. The fairness equation has been defined based on link interference and the required bandwidth to guarantee equitable distribution of network resources among mesh routers and mesh clients. To guarantee the convergence to best global minima, the new generations are generated using the proposed semichaotic techniques with fairness-aware parent selection strategy. The subsequent sections evaluate and validate the proposed fair channel assignment solution.

## 4. Performance Evaluation

In this section, the performance of the proposed channel assignment algorithm is evaluated and compared with related work. The baseline algorithm starts assigning channels to nodes arranged in descending order based on the number of hops to the gateway [17, 21]. This algorithm may not provide a fair allocation of channels in the most sensible area of a WMN, such as nodes far from the gateway. Since the direction of the traffic is towards the gateway, bottleneck problems may occur anywhere in that path, thereby causing network fragmentation, capacity degradation, and the node starvation problem. Python's network library was utilized to implement the simulation of the algorithms presented in this paper.

To illustrate the performance of the proposed algorithm, five performance measures were used, namely, network capacity (NC), the fractional network interference (FNI), per-link capacity, per-link fairness index, and per-link interference index. NC is the total concurrent transmission in the network after the channel assignment algorithm takes place, while FNI is the ratio between network interference and the total conflicting links in the network. FNI is also defined as the number of conflicts that remain after channel assignment relative to the number of conflicts in a single channel network. It is the remaining ratio of interference after applying the channel assignment algorithm. Jain's index [57] has been used to compute the fairness index. Jain's index is independent of the population size, scale, and metric. It is bounded between zero and one where one indicates maximum fairness and zero indicates no fairness. Jain's index is calculated as follows:

(14) $$\begin{array}{c} f\left(X\right)=\frac{{\left[\sum_{i=1}^{n}x_{i}\right]}^{2}}{n\sum_{i=1}^{n}x_{i}^{2}}, \end{array}$$

where *n* denotes the number of links and *x*_*i*_ is the link fairness assigned to the ith link as calculated in (2). It can be noticed from (14) that the fairness *f*(*X*) can be 1 when *x*_*i*_ value is equal for all. This indicates that every link has got fair channel allocation. The network throughput is the sum of the capacity of all links in the network [45, 55, 58]. Link capacity indicator can be calculated from the interference according to the following:

(15) $$\begin{array}{c} \text{link}\_{\text{capacity}}_{l\left(i,j\right)}=\frac{1}{1+{\text{interference}}_{l\left(i,j\right)}},\\ {\text{network}}_{\text{capacity}}\left(\text{NC}\right)=\sum_{\forall \;l\left(i,j\right)\in L}{\text{capacity}}_{l\left(i,j\right)}, \end{array}$$

where *l*(*i*, *j*) is the link between nodes *i* and *j*, interference_*l*(*i*, *j*)_ is the interference on the link *l*_(*i*, *j*)_ after the channel, the assignment has taken place, and *L* is the number of available links in the network.

## 5. Experimental Setup

To analyse the impact of interference between the links, different scenarios were simulated, each of which has a different number of links, namely 5, 16, 36, 46, 58, 78, 119, and 126. With each scenario, many random topologies were generated and tested. All parameters used in this experiment were chosen according to the common practice in the field of channel assignment algorithms.  Table 2 shows the parameters used in the simulation.

The interference model was used to estimate the interference between the links as follows. Two links interfere with each other if they have been set to the same channel and the distance between any two nodes that form the links is smaller than the interference range (514 m). The number of links was created autonomously to preserve network connectivity. That is, the links are created if the distance between the nodes is smaller than the communication range (252 m). For the generalization, in the simulation, the number of nodes, links, and their position have been randomly selected considering the connectivity in mind during node creation. Two versions of the proposed algorithm were evaluated, namely the FA-SCGA-CAA and SCGA CAA. FA-SCGA-CAA is the semichaotic genetic algorithm with a fairness-oriented fitness function which is the main contribution of this paper. Meanwhile, SCGA-CAA is the semichaotic genetic algorithm without a fairness-oriented fitness function. The fitness function used is the interference-based fitness function. In other words, FA-SCGA-CAA aims at maximizing the fairness among nodes while minimizing the link interference, and SCGA-CAA aims at minimizing the sum of link interference.

The proposed FA-SCGA-CAA and SCGA-CAA algorithms have been evaluated by comparing them with Multicriterion Link Ranking CAA (MCLR-CAA) [33] and the Genetic Algorithm Based CAA (GA-CAA) solutions that have been used for channel assignment in several related works [37, 39, 41]. Because solutions in [37, 39, 41] used the original GA algorithm to search for a minimum network interference, this study called this algorithm as IA-GA-CAA interference-aware genetic algorithm-based channel assignment algorithm.

## 6. Results

**Figure 5:**
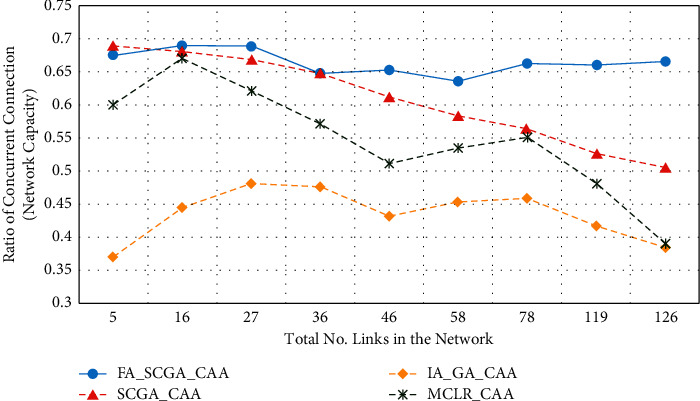
Network capacity (NC).

Extensive simulations were conducted to evaluate the performance of the proposed algorithms and techniques (FA-SCGA-CAA and SCGA-CAA). The results of the proposed algorithm were compared to the multicriterion link ranking-based channel assignment algorithm MCLR-CAA [33] and the interference-aware genetic algorithm-based channel assignment algorithm IA-GA-CAA that was frequently reported in the literature [37, 39, 41].  Figure 5 illustrates the average capacity achieved by the studied channel assignment algorithms. The *x*-axis represents the average number of links in the studied network scenarios, while the *y*-axis represents the corresponding average network capacity. The network capacity is represented by the ratio of the concurrent connection in the network.

As shown in Figure 5, the fairness-oriented semichaotic algorithm FA-SCGA-CAA has the highest network capacity in all simulated scenarios compared with the other studied algorithms. It stays stable at more than 0.65 in most simulated scenarios. The proposed semichaotic algorithm SCGA-CAA achieves higher network capacity than the related metaheuristic genetic algorithm-based CAA (IA-GA-CAA) and the heuristic-based approach MCLR-CAA. MCLR-CAA, however, achieves better network utilization than the metaheuristic algorithm (IA-GA-CAA). This is because the MCLR-CAA considers both interference and link fairness during channel assignment while the IA-GA-CAA tends to minimize the overall network interference without considering the fair distribution of the channels. This explains the improvement gained by the semichaotic approach SCGA-CAA when the MCLR-CAA was utilized to speed up the convergence towards better solutions. The proposed algorithm FA-SCGA-CAA outperforms the other studied algorithms in terms of network utilization. Considering node fairness during channel assignment allows it to improve network performance. Unlike the multicriterion algorithm (MCLR-CAA), the proposed FA-SCGA-CAA algorithm neither biases to specific links based on their criterion nor randomly picks a population that causes the algorithm trapping in local minima. FA-SCGA-CAA starts with a population containing a list of good solutions. Hence, it converges faster, and the results are more effective. It can be concluded that considering the link fairness during channel assignment not only prevents the node starvation problem but also improves the utilization of the available network capacity.

**Figure 6:**
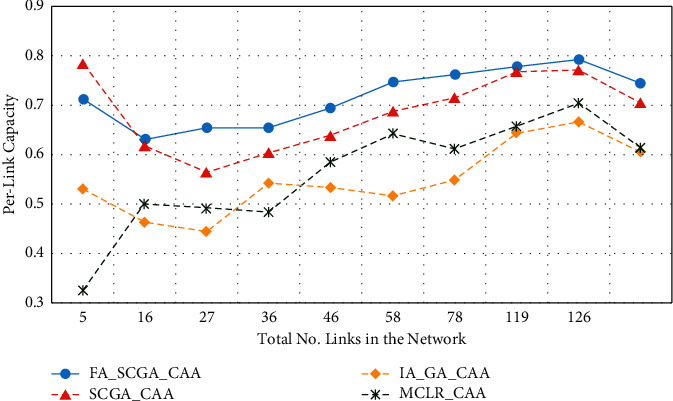
Per-link capacity.

Figure 6 shows the achievements of the studied algorithms in terms of average link capacity. Per-link capacity was calculated using (14) which expresses the capacity degradation due to the interference. The *x*-axis represents the average number of links in 9 scenarios, while the *y*-axis represents the corresponding average link capacity.

From Figure 6, the proposed FA-SCGA-CAA achieves the highest average link capacity in most of the studied scenarios. The average link capacity slightly increases as the number of links increases in the network. It can be seen that the FA-SCGA-CAA outperforms the other studied algorithms under all studied scenarios. The proposed semichaotic-based algorithm (SCGA-CAA) achieves better average link capacity than the multicriterion link ranking algorithm (MCLR-CAA) and the interference-aware genetic algorithm (IA-GA-CAA). Although the FA-SCGA-CAA and SCGA-CAA show close achievements in terms of the average link capacity, they have different achievements in terms of network capacity (see Figure 5). The network capacity of SCGA-CAA drops rapidly as the number of links increases while the network capacity of FA-SCGA-CAA slightly decreases. It can be noticed that in all studied algorithms, the average link capacity increases with increasing the number of links in the network. This can be interpreted as follows. As the network grows bigger, the coverage distance becomes wider which makes the interference smaller. It increases the number of links with nonoverlapping channels, and so does the average link capacity.

**Figure 7:**
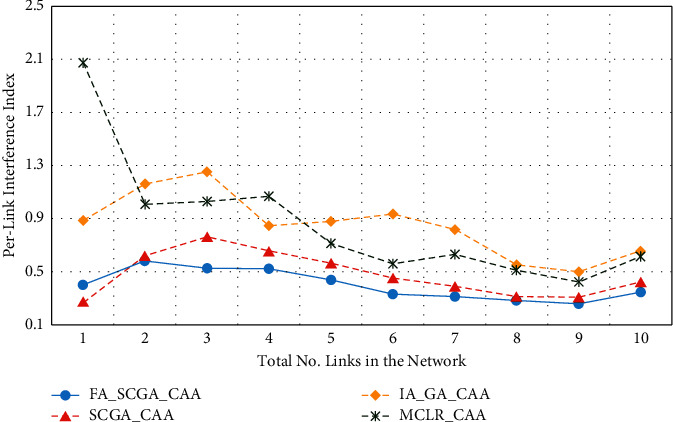
Per-link interference.

Figure 7 illustrates the achievements of the studied algorithms in terms of the average interference index per link. The link interference index is represented by the number of overlapping neighbouring links. The *x*-axis in Figure 7 represents the average number of links of the 9 studied scenarios, while the *y*-axis represents the corresponding average link interference.

As shown in Figure 7, the proposed FA-SCGA-CAA achieved the lowest average link interference among the studied algorithms. It can be noted that in all the studied scenarios, the average link interference decreases as the size of the network increases due to the sparse nature of large networks where the number of links with nonoverlapping channels increases.

**Figure 8:**
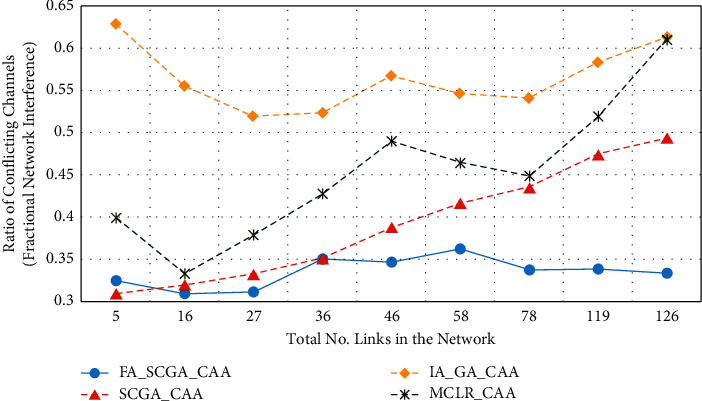
The fractional network interference (FIN).

Figure 8 presents the fractional network interference (FNI). FNI measures the potential number of nodes that are exposed to the starvation problem and calculated based on the number of conflicted channels in the network. In Figure 8, the *x*-axis represents the average number of links in the 9 studied scenarios, while the *y*-axis represents the fractional network interference (FNI).

From Figure 8, it can be seen that the FNI of the proposed FA-SCGA-CAA remains stable below 35% with all studied scenarios. It slightly fluctuates between 30% and 35%, which implies that the ratio of the potential node starvation will be as low as 35% in the worst cases. The FNI of the semichaotic genetic algorithm (SCGA-CAA) and the multicriterion link ranking algorithm (MCLR-CAA) increases as the amount of links increases. Meanwhile, the FNI of the interference-aware genetic algorithm (IA-GA-CAA) remains stable at above 50% in all studied scenarios. This is because IA-GA-CAA does not consider the fair distribution of the channels among available links while it is partially considered in both SCGA-CAA and MCLR-CAA. It can be concluded that the multicriterion-based algorithm (MCLR-CAA) and the semichaotic genetic-based algorithm (SCGA-CAA) can reduce the link interference and thus node starvation.

**Figure 9:**
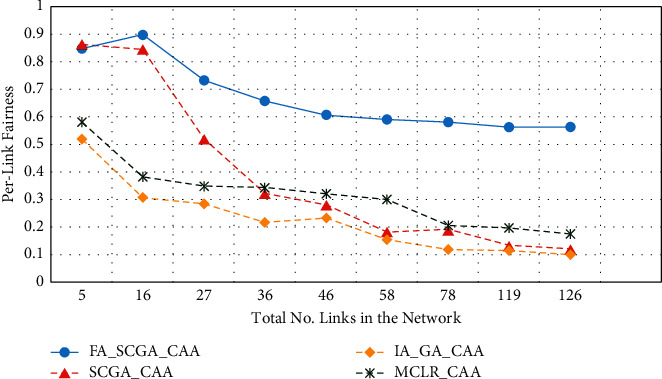
Per-link fairness.

Figure 9 illustrates the achievements in terms of average link fairness. The link fairness is calculated according to (10). Fairness is the ratio between the link data rate after channel assignment and the link capacity before the channel assignment. The fairness index ranges between one (for the highest fair distribution) and zero (for the lowest unfair distribution). The *x*-axis in Figure 8 represents the average number of links in the 9 studied scenarios while the *y*-axis represents average link fairness.

From Figure 9, the proposed FA-SCGA-CAA achieves the highest average fairness index among all studied algorithms and in all the simulated scenarios. The fairness index slightly drops as the number of links increases. Although the fairness index of the semichaotic genetic algorithm (SCGA-CAA) is high when the number of links is low, it rapidly drops from 0.85 to 0.1 when the number of links increases. Meanwhile, the fairness indexes of the MCLR-CAA and IA-GA-CAA are low and drop lower than 0.2 when the network grows bigger.

**Figure 10:**
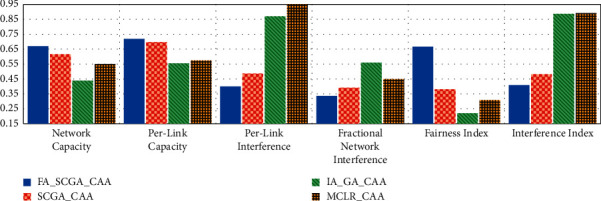
Summary of the overall performance of the proposed FA-SCGA-CAA and related work.

From Figures 5–9, it can be concluded that the developed fairness fitness functions that maximize fairness and minimize the interference not only prevent node starvation problem but also improve the utilization of network capacity.  Figure 10 presents a comparative summary of the performance of the proposed FA-SCGA-CAA and the SCGA-CAA, and the related works MCLR-CAA and IA-GA-CAA. The *x*-axis represents the performance measures, namely, the average of the network capacity in terms of concurrent connection, the average of potential starvation nodes in terms of the ratio of the conflicting channels or the fractional network interference (FNI), the average link fairness index, and network interference index. The *y*-axis represents the average value of the corresponding performance measures.

As shown in Figure 10, the proposed FA-SCGA-CAA outperforms the other studied algorithms for every analysed performance measure. In terms of network capacity, the FA-SCGA-CAA algorithm improves the utilization of the network capacity by 23% compared to IA-GA-CAA. Similarly, the per-link capacity was improved by 16.9% compared to IA-GA-CAA. In terms of per-link interference, the proposed FA-SCGA reduces the interference by 46.4%. Likewise, FA-SCGA-CAA also reduces the fractional network interference by 22% as compared to IA-GA-CAA. The fractional network interference has a direct relationship with the potential nodes' starvation. In terms of average link fairness, the proposed FA-SCGA-CAA algorithm improves the fairness by 44% compared to the fairness unaware approach IA-GA-CAA. In terms of interference, 46% reduction is achieved by the FA-SCGA-CAA as compared to the IA-GA-CAA.

## 7. Discussion

This section presents the discussion of the results presented in the previous section. The study aims at designing and developing a Fairness-Oriented Semichaotic Genetic Algorithm-Based Channel Assignment Technique (FA-SCGA-CAA) that addresses the node starvation problems, through fair (equitable) channel distribution among mesh nodes in the WMN. A fair distribution of channel assignment should not end up with a node starvation problem. However, existing channel assignment schemes suffer two main drawbacks as follows. Firstly, existing solutions focus on minimizing the total interference to achieve effective utilization of network resources. However, such a solution leads to a node starvation problem where the throughput of some links drops lower than the minimum required throughput. That is, reducing the total interference does not necessarily ensure equal distribution of channels in the network. As shown in Figure 5, reducing total network interference leads to unfair distribution of the available network bandwidth and thus lower network capacity. The proposed FA-SCGA-CAA algorithm distributes the channel interference based on the required bandwidth by each link. That is, links that require higher bandwidth are given the channel with lower interference. Thus, nodes do not suffer from the starvation problem due to the consideration of the minimum required bandwidth for each link. Accordingly, the total of concurrent links has been increased which interprets the results obtained by the proposed FA-SCGA-CAA algorithm (see Figure 5). The results in terms of per-link capacity in Figure 6 support the study hypothesis regarding how the total network capacity is improved by achieving link-level fairness based on the minimum required bandwidth. Consequently, the results in terms of per-link interference in Figure 7 and the fractional network interference interpret the improvement gained in both per-link capacity and the overall network capacity. The proposed fairness-oriented algorithm guarantees the minimum required capacity for each link which contributes to avoiding node starvation problems. The per-link fairness that is depicted in Figure 9 suggests that as the individual link fairness index increases, the fairness of the nodes using that link increases as well which interprets how the proposed algorithm approached the node starvation problem. Comparing with the related studied algorithms, IA-GA-CAA [37, 39, 41] and MCLR-CAA [33] in which the fairness was represented in terms of total interference, the equitable distribution of the resources among all nodes is the ultimate goal of the proposed algorithm FA-SCGA-CAA to resolve the node starvation problem. The proposed algorithm FA-SCGA-CAA distributes the resources based on the minimum required bandwidth for each node, which is not necessarily equal as was considered by the extant studies. Comparison with IA-GA-CAA shows that the proposed FA-SCGA-CAA reduced the potential node starvation by 22% and improved network capacity utilization by 23% (see Figure 10).

The second drawback of the existing GA-based solutions [37, 39, 41] such as the IA-GA-CAA algorithm is that the population samples are randomly generated which leads to the premature solution since the search is usually directed to local minima. The proposed FA-SCGA-CAA algorithm avoids such problems by introducing the concepts of a semichaotic approach for creating a population. The proposed semichaotic GA-based technique creates the primary chromosome with powerful genes based on multicriterion links ranking channel assignment algorithm. Such a chromosome was used with a proposed semichaotic technique to create a strong population that directs the search towards the global minima. The proposed semichaotic was also used during the mutation and parent selection of the new genes. To show the impact of the proposed semichaotic techniques in the performance of the proposed algorithm, the SCGA-CAA algorithm is implemented. The difference between the FA-SCGA-CAA and SCGA-CAA is that FA-SCGA-CAA includes the fitness function in (11). This fitness function is to calculate the link fairness based on the achieved bandwidth considering the interference resulted from the channel assignment. As shown in Figures 6–10, the proposed SCGA-CAA algorithm improves capacity while reducing the interference as well as achieving a better fairness index and lower interface index compared with the IA-GA-CAA. These results prove that the semichaotic technique succeeds in selecting strong populations and directs the search towards the global minima effectively. The proposed fitness functions in the proposed FA-SCGA-CAA algorithm help in selecting the best solution among the available solutions generated by the GA which interprets why the proposed FA-SCGA-CAA outperforms the SCGA-CAA.

**Figure 11:**
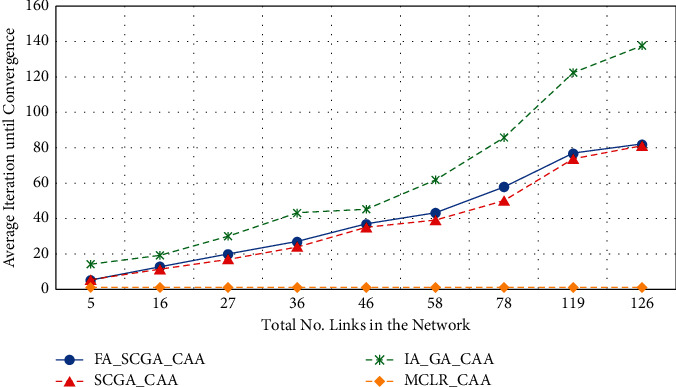
The average iterations until convergence.

Besides improving the effectiveness of the network, the proposed FA-SCGA-CAA and SCGA-CAA algorithms improve the efficiency of the channel assignments in terms of fast convergence to find the best solution. Furthermore, the cost in terms of time complexity of the studied metaheuristic algorithms [37, 39, 41] including the proposed FA-SCGA-CAA and SCGA-CAA can be represented in terms of big-O notations. The time complexity metaheuristic algorithms, FA-SCGA-CAA, SCGA-CAA, and IA-GA-CAA, are *O*(gnm), where *g* is the number of generations (number of the iterations needed until convergence), *n* is the number of links (number of genes), and *m* is the population size. The time complexity of the MCLR-CAA is*O*(*n*), where *n* is the number of links in the network. Although the MCLR-CAA is more efficient in terms of time complexity than the metaheuristic algorithms, it has lower performance compared with the proposed model in terms of network capacity, interference, and fairness which are essential for WMNs. That is, in WMN the effectiveness of the channel assignment algorithms in terms of improving network capacity by reducing the interference among the channels while achieving fairness is more important. However, the efficiency of the proposed FA-SCGA-CAA and SCGA-CAA algorithms evaluated in terms of the number of iterations is needed until finding the best solution.  Figure 11 illustrates the performance gained by applying the semichaotic approach in speeding up the convergence rate of the genetic algorithm. The *x*-axis represents the average number of links in the 9 studied scenarios while the *y*-axis represents the average iterations until convergence.

From Figure 11, the proposed semichaotic method has improved the speed rate of the convergence as compared to the original genetic algorithms. This is because the semichaotic method characterized the selected search space by more powerful genes. Hence, selecting a powerful primary chromosome leads to create a population with powerful genes that speeds up the convergence. Furthermore, the multicriterion method that is used to create the primary chromosome considers both the fairness and the interference during link ranking. Thus, it directs the convergence towards the global optima. This explains the improvements gained by the proposed FA-SCGA-CAA which employs both the fairness-based fitness function and the semichaotic approach to create the primary chromosome and search for the most effective solution.

## 8. Conclusions

In this paper, the Fairness-Oriented Semichaotic Genetic Algorithm-Based Channel Assignment Technique (FA-SCGA-CAA) for wireless mesh network was proposed. The node starvation problem that was overlooked by the extant studies due to unfair channel assignment has been addressed in this study. The unfair distribution of the network resources of the existing solution has been attributed to two main drawbacks. Firstly, the equal distribution of the interference on the network does not lead to a fair distribution of network resources. Second, randomly generating the population samples in the genetic algorithm-based solutions leads to a premature solution where the search is usually directed to local minima. These two limitations have caused unfair distribution of the channels and thus node starvation problem in WMN. To achieve node fairness, a semichaotic genetic algorithm-based technique was proposed to create a diverse population with informative features that converge to the best solution and avoid being trapped in the local minima. The channel assignment problem was formulated as an optimization problem with a fairness-aware fitness function. The fairness-oriented fitness function combines several factors that represent the network topology, load status, and required bandwidth/throughput in one function. The fairness was defined on node level to address the node starvation problem. Extensive experimental evaluations were conducted to measure the performance of the proposed technique and compare it with existing solutions. The results showed that the proposed algorithm outperformed existing solutions in terms of improving the link fairness and utilization of network capacity while reducing the interference and the potential number of node starvations. It can be concluded that the proposed FA-SCGA-CAA is reliable to maintain high node-level fairness while maximizing the utilization of the network resources, which is the ultimate goal of many wireless networks. Similar to any genetic-based algorithm, the proposed algorithm has two main drawbacks as follows. The first issue is that setting up the number of iterations is scenario specific. Therefore, it is difficult to select a fixed number of iterations for generalization. The second issue is on the selection of the convergence threshold. These issues contributed to the overall link fairness. We are currently working on addressing those two issues and the new findings will be the subject of our next publication.

## Figures and Tables

**Algorithm 1:**
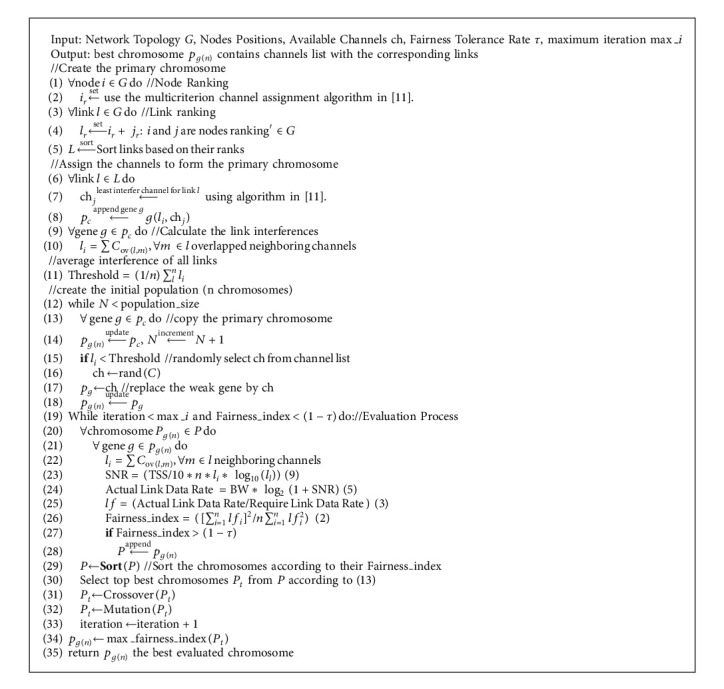
The proposed FA-SCGA-CAA algorithm and the pseudocode of the proposed channel assignment algorithm FA-SCGA-CAA.

**Table 1:** Symbols description.

Symbol	Description
*P*, *P*_*t*_	*P*, parents (old generation) and *P*_*t*_ children (new generation)
*p*_*c*_, *p*_*g*(*n*)_	*p*_*c*_is the primary chromosome and *p*_*g*(*n*)_ is the selected chromosome
SNR	Signal-to-noise ratio
*i*_*r*_, *j*_*r*_	The ranks of nodes *i* and *j*
*τ*	Tolerance rate
*l*_*i*_	The interference on the link *i*

**Table 2:** Simulation parameters.

Parameter	Configuration
Propagation model	Free space/two ray ground
Antenna	Omnidirection
MAC type	802.11a/b
Orthogonal channels	12/3
Communication range	252 m
Interference distance	514 m
Number of nodes	Varies from 50 to 200
Number of links	Varies from 10 to 130
Number of radios	3 for each mesh router
Connectivity degree	3
Simulation area	1000 m × 1000 m

## Data Availability

Data will be made available upon request to the corresponding author.
